# Integrated liver transcriptomic data reveal differences in aging-associated regulation between metabolic dysfunction-associated steatotic liver disease and normal liver aging

**DOI:** 10.1371/journal.pone.0356820

**Published:** 2026-08-21

**Authors:** Tanchun Wang, Qin Huang, Yan Wang, Cong Li, Juan Ni, Fang Xie

**Affiliations:** 1 Department of Liver Disease, Jinling Hospital, Affiliated Hospital of Medical School, Nanjing University, Nanjing, China; 2 Nursing Department, Jinling Hospital, Affiliated Hospital of Medical School, Nanjing University, Nanjing, China; 3 Department of Integrated TCM and Western Medicine, Nanjing Hospital Affiliated to Nanjing University of Chinese Medicine, Nanjing, China; University of Diyala College of Medicine, IRAQ

## Abstract

**Background:**

Age-related molecular trajectories of metabolic dysfunction-associated steatotic liver disease (MASLD) remain insufficiently characterized, limiting age-stratified risk assessment and intervention.

**Objective:**

To test whether MASLD-related molecular changes with age merely reflect accelerated physiological aging or represent a disease-specific divergence.

**Methods:**

We utilized public transcriptomic data from normal liver and MASLD samples to identify differential transcriptomic signals. We employed Spearman correlation analysis and generalized additive models (GAM) for nonlinear age-related trajectory modeling, and functional enrichment analysis for signaling pathway characterization.

**Results:**

We integrated 1,354 public liver transcriptome samples from GEO and GTEx databases to construct a cross-age cohort. Differential expression and age-correlation analyses showed minimal overlap (only 22 genes) between age-associated genes in MASLD and controls, with some genes displaying opposite age-related trends. GAM identified four major expression patterns in MASLD—stable, early-life change, late-life acceleration, and mid-life fluctuation—with trajectory inflection time points around ages ~35 and ~75. Functional enrichment indicated that control age-associated genes mainly involved classical cell cycle processes, whereas MASLD changes were enriched for protein deubiquitination, proteostasis imbalance, and p53-related stress and apoptotic signaling. Many MASLD age-associated genes were also associated with fibrosis stage or NAS, largely in concordant directions.

**Conclusion:**

MASLD exhibits disease-specific age-related transcriptional remodeling characterized by dysregulation of proteostasis and deubiquitination pathways, activation of cellular stress-response programs, and enrichment of p53-associated apoptotic signaling, rather than a simple acceleration of physiological aging.

## Introduction

MASLD (previously known as non-alcoholic fatty liver disease, NAFLD) has become the predominant public liver disease health burden globally [1–3]. Increasing age is closely associated with the risk of adverse outcomes of MASLD. Older individuals are more prone to progressing to steatohepatitis, significant fibrosis, hepatocellular carcinoma (HCC), and other related complications [4–6].

Age not only reflects long-term accumulated risks and an increased burden of comorbidities, it also corresponds to the comprehensive clinical effects such as genetic instability, higher level of inflammation, lower liver regenerative capacity, worse immune microenvironment and cellular stress tolerance [7–9]. Therefore, the progression of age in MASLD likely interacts with the liver’s molecular phenotype and pathological evolution, thereby increasing a heterogeneous risk profile across different age stages [10–13].

Existing age-related research in MASLD has employed cross-sectional comparisons to examine differences in prevalence, incidence, fibrosis burden, and adverse clinical outcomes across disease stages in older adults [5,11,12]. Mechanistically, aging is linked to progression via cellular senescence, mitochondrial dysfunction, impaired autophagy, chronic inflammation, and dysregulated nutrient-sensing [14,15]. While recent multi-omics studies highlight molecular heterogeneity [15,16], most focus on specific age strata or individual mechanisms rather than modeling transcriptomic changes along a continuous lifespan axis. Consequently, studies systematically tracking age-related molecular trajectories in MASLD versus physiological liver aging remain limited [15,17–19].

It remains debated whether age-related changes in MASLD simply accelerate physiological aging or represent a disease-specific divergence. Specifically, does aging merely amplify intrinsic biological decay, or does chronic metabolic stress induce unique molecular shifts, potentially driving certain pathways in opposite directions [12,20–22]? Resolving this distinction is critical for understanding MASLD heterogeneity and developing age-stratified clinical strategies [23–25].

Aging biology typically identifies mitochondrial dysfunction, telomere shortening, cellular senescence, decreased proteostasis, and chronic inflammation as multi-tissue hallmarks [26,27]. However, the liver’s central metabolic roles—including lipid and bile acid metabolism, detoxification, and protein synthesis [28–30]—predispose it to pronounced endoplasmic reticulum stress, altered autophagic flux, and ubiquitin-proteasome pressure under chronic metabolic stress [31–33]. Consequently, we hypothesize that age-related remodeling in MASLD is not a simple extension of classical aging, but a pathological reorganization driven by metabolic stress and protein quality control [10,25,34]. Validating this requires systematic testing across large, multi-cohort samples spanning a broad age range [23].

Public transcriptomic data is ideal for aging research, offering comprehensive molecular characterization, larger sample sizes, and greater statistical power than single-cohort studies [35,36]. However, since age-related biological processes are often non-linear, traditional linear regression may obscure phase-specific accelerations, threshold effects, or critical transitions [37–39]. In MASLD, the mid-life decline in metabolic elasticity, accumulation of comorbidities, and late-life shifts in regenerative capacity and immune surveillance can drive non-linear transcriptomic fluctuations [40,41]. Therefore, statistical tools capable of modeling smooth, non-linear trajectories are methodologically essential, which holds significant clinical value for identifying critical transition intervals and linking them to underlying mechanistic modules [42]. Under this background, we integrated multi-center liver transcriptome data from Gene Expression Omnibus (GEO) database and Genotype-Tissue Expression (GTEx) project to construct a cross-age cohort. Differential expression analysis showed that MASLD retained core transcriptional changes across age groups, while the number of altered genes increased with age. Age-correlation analysis further showed minimal overlap between age-related genes in MASLD and controls, with some shared genes displaying opposite trends. GAM analysis revealed non-linear expression changes, with major transition intervals around ages ~35 and ~75. Functional enrichment further suggested that age-related changes in MASLD were more related to protein homeostasis and stress responses, rather than the canonical patterns of physiological aging. Together, these findings suggest that MASLD shows specific age-related transcriptional alternation, rather than simply accelerated normal liver aging.

## Methods

### Data collection, screening, and integration

Liver sample search was performed in the GEO database using ‘liver’ as the keyword. After obtaining the initial study series, filtering was conducted based on criteria including human species, liver tissue origin, and high-throughput transcriptome sequencing platforms. In addition, liver samples were further included from the GTEx database. Subsequently, through manual review of GEO metadata and original literature, studies involving drug interventions, unclear clinical backgrounds, or excessively small sample sizes were excluded. High-quality samples comprising healthy control groups and MASLD groups were integrated. Data processing was primarily performed using R Studio. During data merging, for GEO data, the uniform count matrices generated by NCBI were used, and gene names were uniformly converted to gene symbols based on the NCBI human gene annotation table. For GTEx data, the V10 expression matrix was used and the corresponding gene names were extracted. By matching metadata, alignment of gene names and expression data for all samples was ensured.

### Data preprocessing and batch effect reduction

We removed non-target gene types such as rRNA and pseudogenes, retaining only protein-coding genes. The filterByExpr function from the edgeR package was used to filter low-expression genes [43]. To address potential batch effects present in multiple datasets, the ComBat_seq function from the sva package was employed [44]. This approach helps reduce batch effects in the raw data originating from different sources by using the dataset source as a covariate to correct the raw count matrix.

### Differential expression analysis (DEA)

DEA was performed using the DESeq2 package [45], a generalized linear model based on the negative binomial distribution. The screening criteria for significantly differentially expressed genes (DEGs) were set as a Benjamini-Hochberg (BH) adjusted *P*-value (*padj*) less than 0.05 and an absolute logarithmic fold change (|log2FC|) greater than 1.

### Age-expression correlation analysis

In this analysis, age was obtained from the sample metadata, recorded in years, and specified as a continuous explanatory variable, whereas the VST-transformed expression value of each gene was treated as the dependent variable. To quantify the trends of gene expression levels with increasing age, we used variance stabilizing transformation (VST) data through DESeq2. The cor.test function was used to calculate Spearman’s rank correlation coefficients (rho) between gene expression values and the continuous age variable. The BH method was used to correct the *P*-values from the correlation analysis for multiple hypothesis testing. Based on the regulation directionality and significance of the correlation coefficients, genes were classified into four dimensions for synergistic or antagonistic analysis.

### Non-linear expression trajectory modeling based on GAM model

We used the mgcv package to fit GAM for characterizing age-related expression trajectories [46]. VST-transformed gene expression was treated as the dependent (outcome) variable, whereas age was treated as a continuous explanatory variable. For each gene, VST-transformed expression was used as a smooth function of age using a thin plate regression spline, with the basis dimension set to 4 to limit model complexity and reduce overfitting. We simultaneously extracted the effective degrees of freedom (edf) of the smooth term as a measure of non-linearity. For descriptive classification, we evaluated the magnitude and direction of change across different age intervals based on the model’s predicted expression curves. To identify age-related transition points, we used the gratia package to estimate first derivatives of the GAM smooth terms across 500 age grid points using central finite differences, with simultaneous 95% confidence intervals. For genes with significant GAM results, transition ages were defined as points where the first derivative changed sign and were estimated by linear interpolation between adjacent age grid points.

### Functional enrichment analysis

Functional annotation and enrichment were implemented using the clusterProfiler package [47]. The bitr function was used to convert gene symbols to Entrez IDs. The enrichGO function was used to perform enrichment analysis for Gene Ontology biological processes (GO-BP). The significance threshold was uniformly set at an adjusted *P*-value (*padj*) less than 0.05 using BH correction.

### Clinicopathological association analysis

We collected clinical phenotypes related to MASLD severity. To maximize statistical power, analyses were limited to fibrosis stage and NAFLD activity score (NAS), which were available in the largest number of datasets. Fibrosis stage ranged from F0 (no fibrosis) to F4 (cirrhosis), and NAS ranged from 0 to 8, with higher scores indicating greater histological activity. Associations between MASLD age-associated gene expression and fibrosis stage or NAS were assessed using regression models adjusted for age and dataset source, with FDR < 0.05 considered statistically significant.

## Results

### Multi-center liver transcriptome data collection and filtering

**Fig 1:**
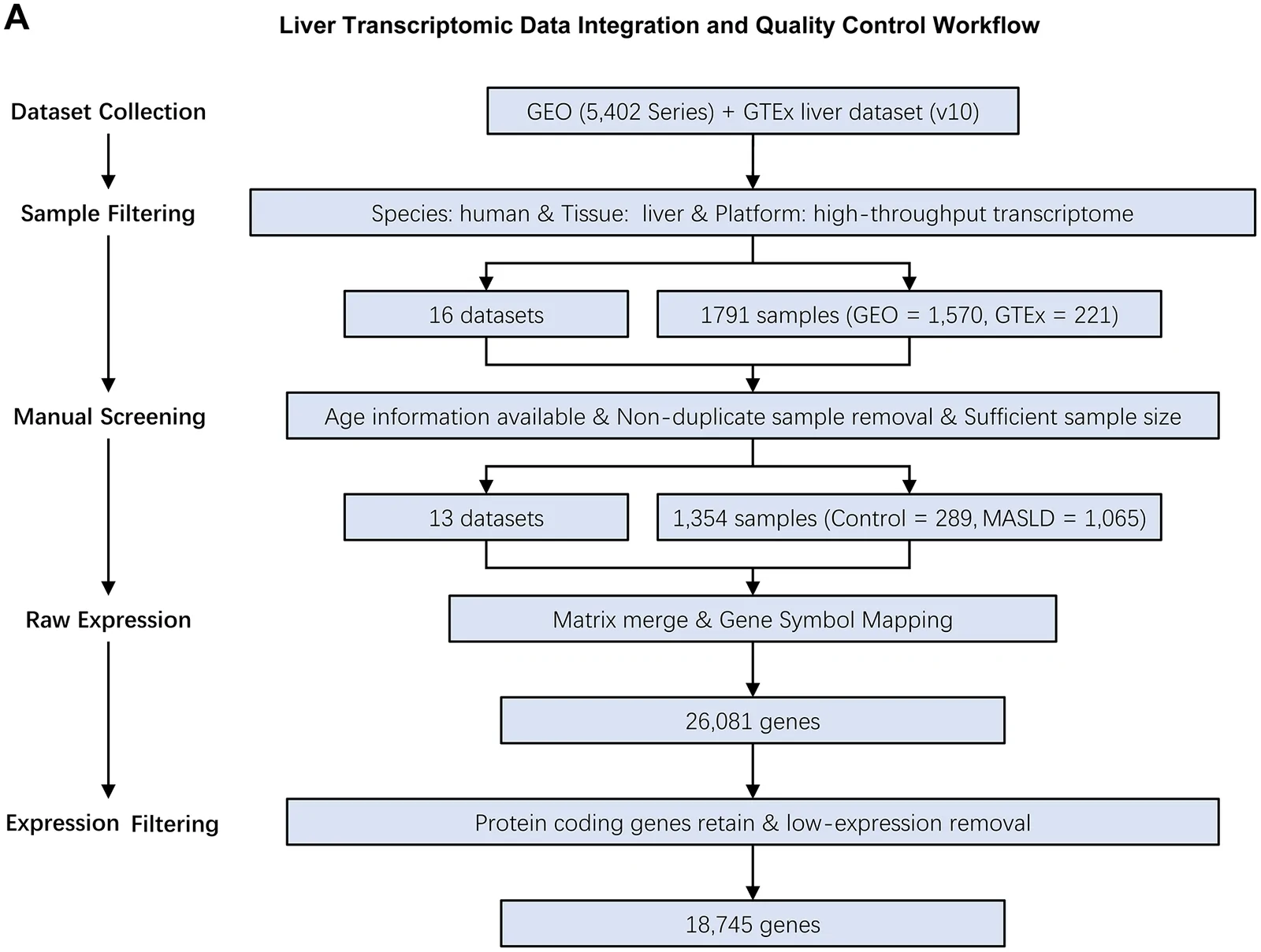
Workflow of liver transcriptomic data collection and filtering. (A) Through retrieval from the GEO and GTEx databases, multi-criteria filtering, and manual review, a high-quality liver transcriptome dataset was integrated.

First, 5,402 liver-related series were retrieved from GEO, and after multi-criteria filtering based on species, tissue, and platform, 1,791 samples were initially obtained. Through manual metadata review, 13 datasets comprising 1,354 high-quality samples (289 healthy controls and 1,065 MASLD; age range 9–92 years) were selected (S1 Table). Upon merging raw expression matrices, noise genes (mitochondrial and ribosomal) and low-expression genes were removed, reducing the gene count from 26,081–18,745 (Fig 1A). To address significant batch effects and expression inconsistencies across datasets, we employed ComBat_seq for correction. After quality control, the expression distributions were aligned at the median level and PCA showed a marked reduction in dataset-specific clustering, making the dataset suitable for subsequent differential expression analysis (DEA).

### Identification of DEGs in the global MASLD population and age-stratified groups

**Fig 2:**
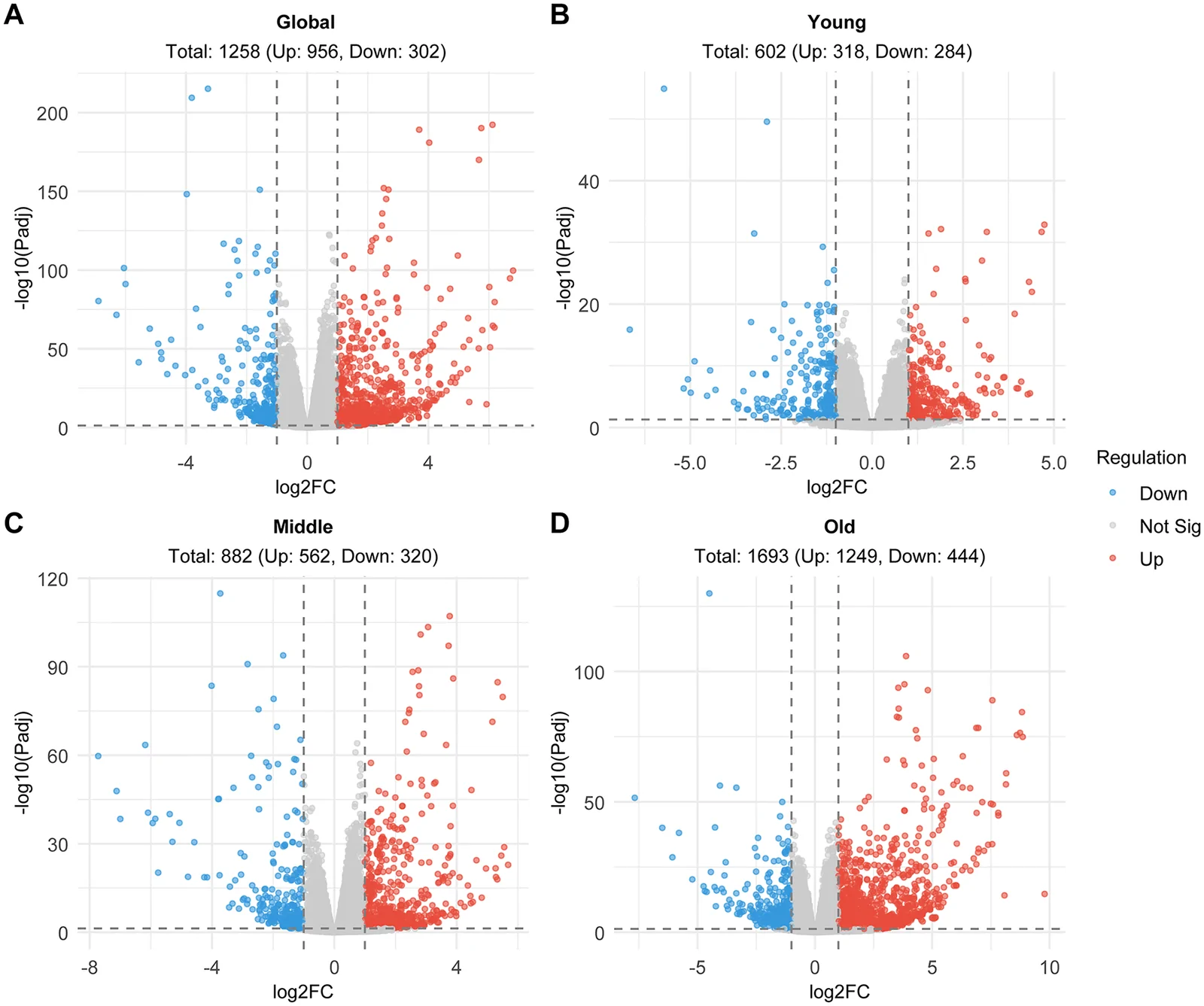
DEA between MASLD and controls in the overall cohort and across age groups.A-D: Volcano plots of DEA for the four sample groups. (A) Global group. (B) Young group. (C) Middle-aged group. (D) Elderly group. Red indicates significantly upregulated genes, and blue indicates significantly downregulated genes.

DEA was performed using DESeq2, with DEGs defined by *padj* < 0.05 and |log2FC| > 1 (S2 Table). We first compared MASLD with healthy controls from the global population. A total of 1,258 DEGs were identified, including 956 upregulated and 302 downregulated genes (Fig 2A).

Subsequently, all samples were stratified into three age groups including young (≤39 years), middle-age (40–59 years), and old-age (≥60 years). The young group comprised 282 samples (54 controls and 228 MASLD), the middle-aged group comprised 710 samples (154 controls and 556 MASLD), and the elderly group comprised 362 samples (81 controls and 281 MASLD).

DEA between MASLD and controls was then performed within each age group. In the young group, 602 DEGs were identified (318 upregulated and 284 downregulated), in the middle-aged group, 882 DEGs were identified (562 upregulated and 320 downregulated), and in the elderly group, 1,693 DEGs were identified (1,249 upregulated and 444 downregulated) (Figs 2B-D).

### Quantitative trends and intersection analysis of DEGs across age strata

**Fig 3:**
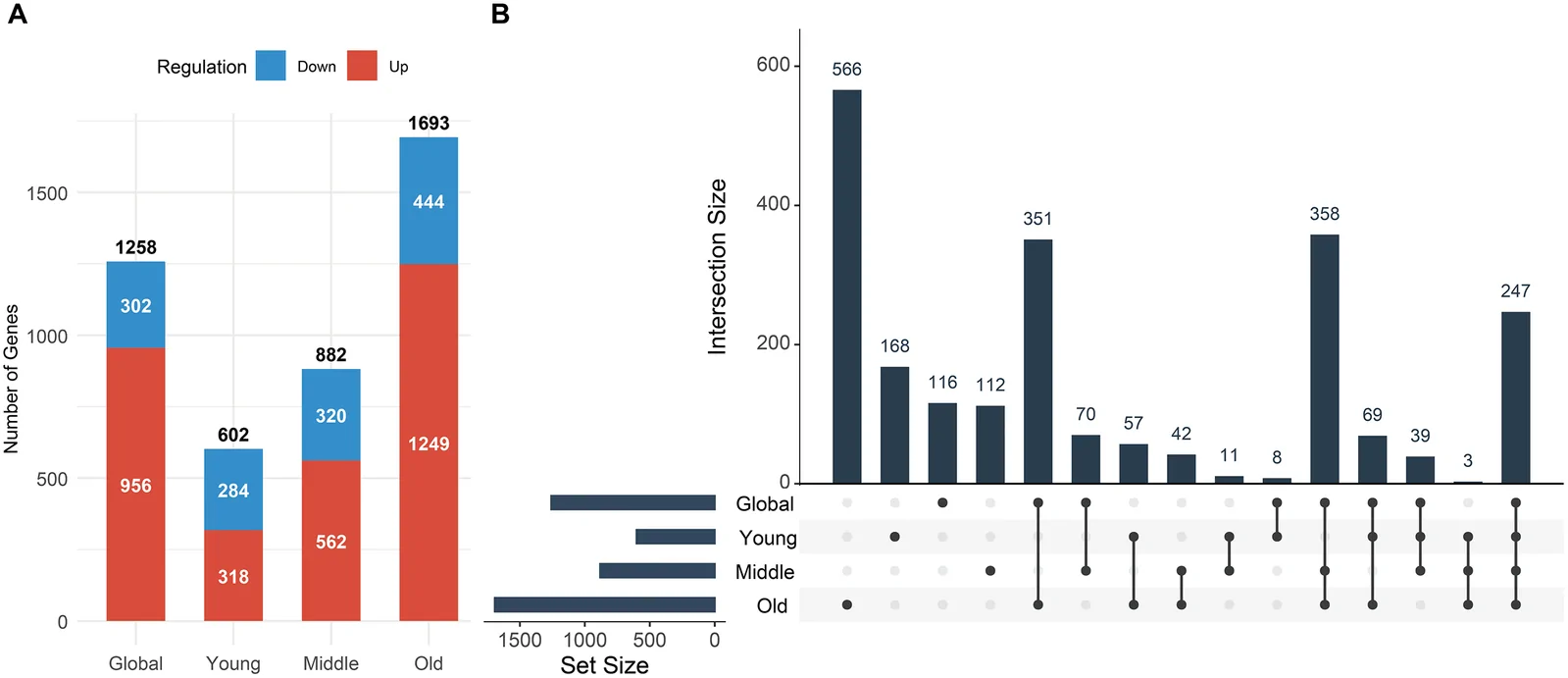
Distribution and overlap of DEGs across age groups. (A) Bar plot showing the total number of DEGs and the distribution of upregulation and downregulation in each group. (B) Upset plot illustrating the intersections and unique genes among different groups.

In both the global population and age-stratified group analyses, upregulated genes were consistently more than downregulated DEGs. Notably, the total number of DEGs increased with age (Fig 3A). To further assess shared and unique transcriptional changes, we compared DEG overlaps between the global analysis and each age stratum. The UpSet plot showed that 247 common DEGs were shared across the global analysis and all three age groups, accounting for 19.6% of global DEGs, 41.0% of young-group DEGs, 28.0% of middle-aged-group DEGs, and 14.6% of elderly-group DEGs (Fig 3B). These genes represent relatively stable transcriptional signatures of MASLD across ages.

In addition, the numbers of DEGs shared only between the global analysis and each age group increased with age, with 8 (1.3%), 70 (7.9%), and 351 (20.7%) DEGs in the young, middle-aged, and elderly groups, respectively. Regarding age-stratum-specific DEGs (only significant within the group), the elderly group had the most, with 586 (34.6%), followed by the young group with 168 (27.9%) and the middle-aged group with 112 (12.7%). Additionally, 116 DEGs (9.2%) were uniquely identified in the global analysis. Together, these findings suggest that MASLD retains a stable core transcriptional signature across age groups, while also showing age-related heterogeneity, with additional age-specific transcriptional alterations emerging during aging.

### Age-associated transcriptional analysis within the MASLD population

**Fig 4:**
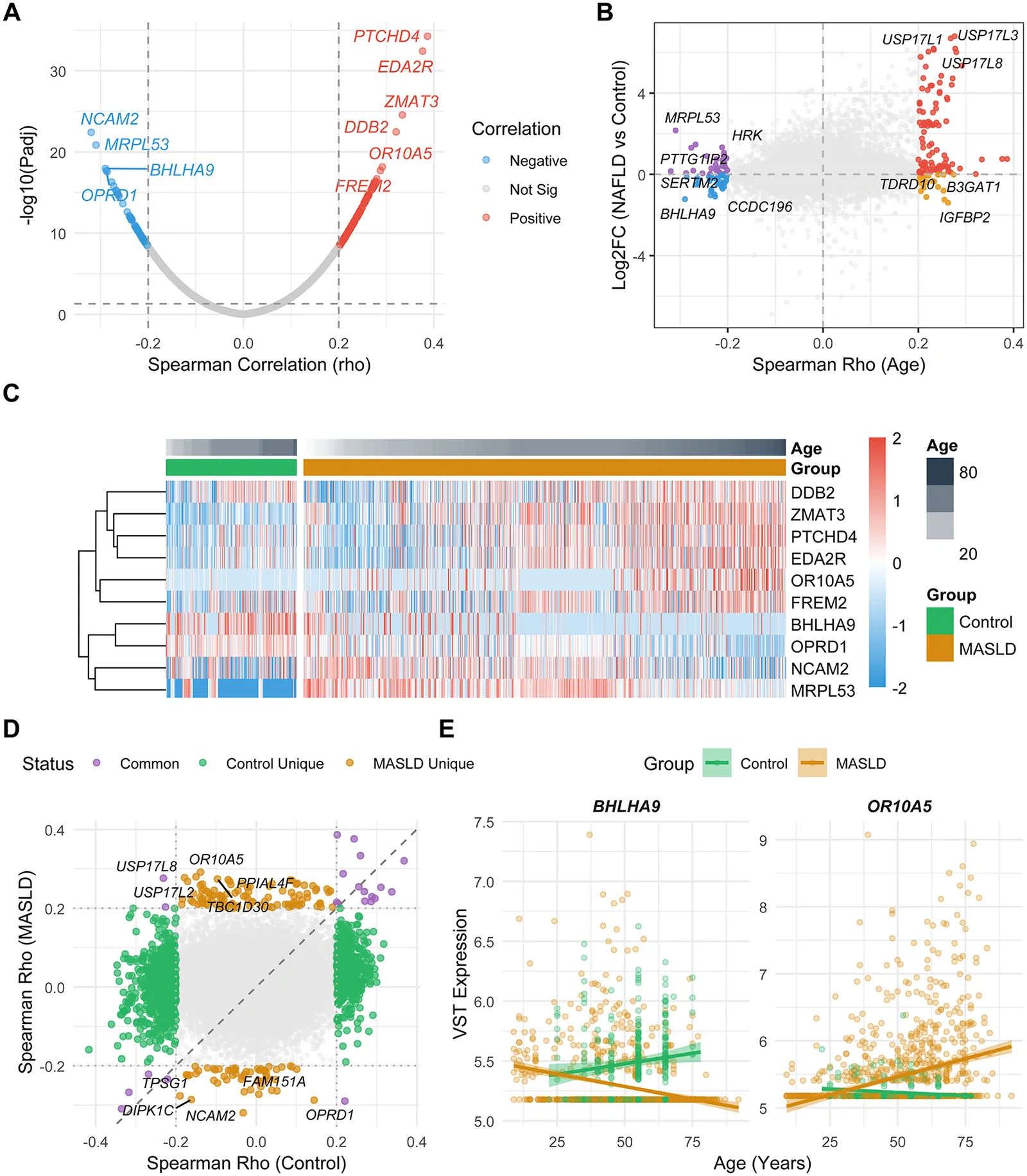
Identification and characterization of age-associated genes in MASLD. (A) Scatter plot showing genes significantly correlated with age in MASLD samples. (B) Scatter plots displaying the four-quadrant classification based on differential expression (log2FC) and age correlation (rho). (C) Heatmap showing expression patterns of the top 10 genes with the strongest age correlation. (D) The comparison and intersection of age-associated genes between control and MASLD groups. (E) Two examples of overlapping age-associated genes exhibiting opposite trends between control and MASLD groups.

To investigate age-related regulatory trends within the MASLD population, we extracted all MASLD samples and performed Spearman correlation analysis. Using a significance threshold of *padj* < 0.05 and |rho| > 0.2, we identified 180 genes significantly correlated with age (Fig 4A), including 121 positively correlated and 59 negatively correlated.

By integrating the log2FC values of these genes from the global DEA of MASLD versus control samples, we classified them into two synergistic categories: 99 upregulated with positive correlation and 32 downregulated with negative correlation, and two antagonistic categories: 27 upregulated with negative correlation and 22 downregulated with positive correlation (Fig 4B).

Based on correlation degree, we displayed the top 10 representative genes: USP17L3, USP17L8, and TDRD10 showed positive correlation with upregulation; B3GAT1 and IGFBP2 showed positive correlation but downregulation; MRPL53, HRK, and PTTG1IP showed negative correlation with upregulation; SERTM2 and BHLHA9 showed negative correlation with downregulation (Figs 4A-D).

Additionally, 981 age-associated genes were identified in the control group, however, only 22 of them overlapped with those identified in the MASLD group (Fig 4D). Among these overlapping genes, expression trends were not entirely consistent. For example, BHLHA9 decreased with age in MASLD but increased in controls, and OR10A5 exhibited the opposite trend (Fig 4E). These findings suggest that a considerable portion of age-associated alterations in MASLD cannot be simply explained by physiological aging factors and may instead reflect MASLD-based age-related regulation.

### Trajectory classification of age-associated genes in MASLD and key age-related transition intervals

**Fig 5:**
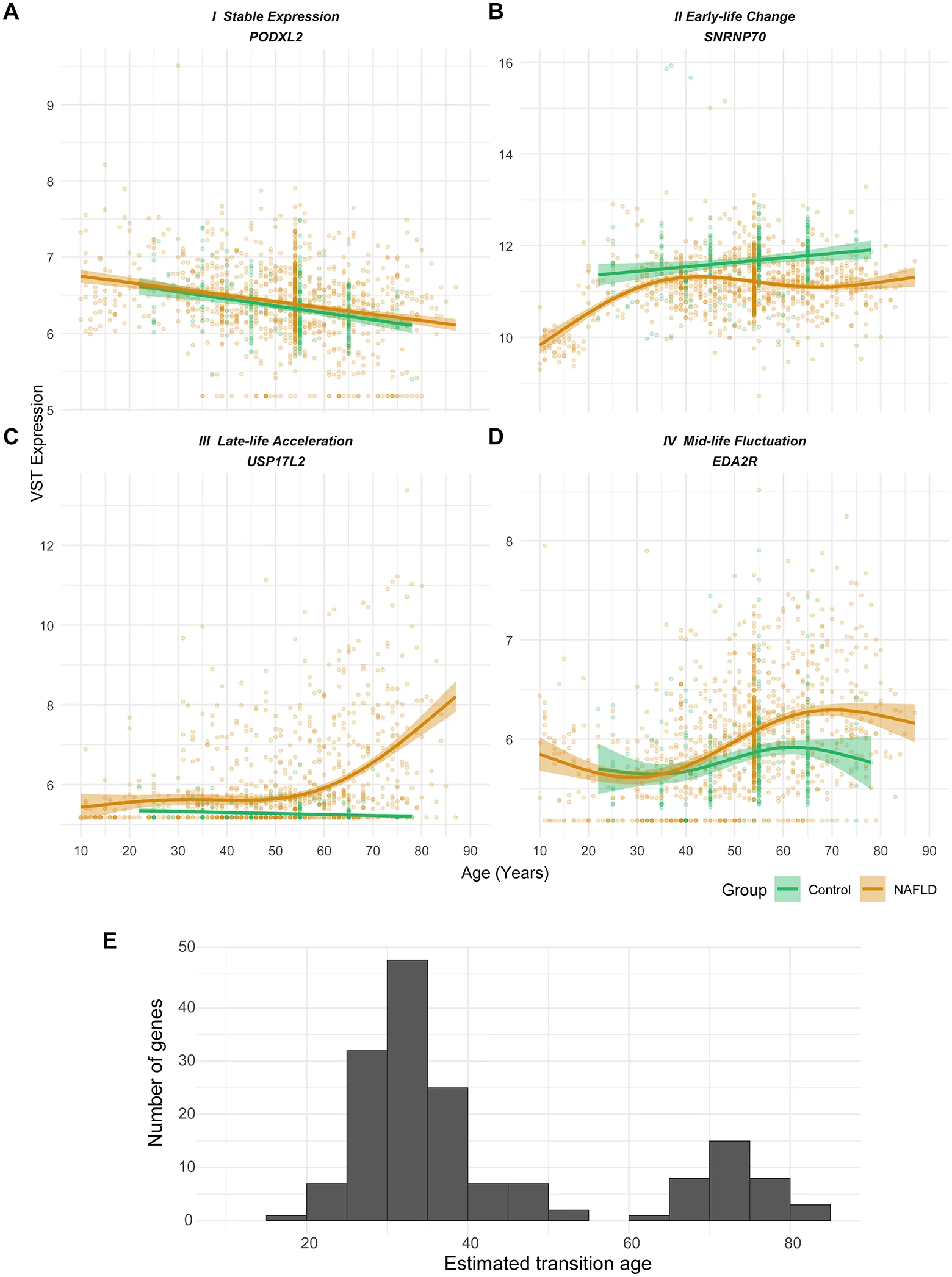
GAM-based classification of age-related expression trajectories in MASLD. (A) Class I: Stable expression type; (B) Class II: Early- life changing type; (C) Class III: Late-life accelerating type; (D) Class IV: Mid-life fluctuating type. (E) Estimated transition age and critical window.

Recognizing that transcriptional expression may exhibit nonlinear fluctuations across young, middle-aged, and elderly stages [48], we further applied GAM to fit and classify the age trajectories of the 180 age-associated genes identified in MASLD. This model aimed to capture stage-specific characteristics that linear correlations might fail to reveal. Based on the overall morphology of GAM-predicted curves and the intensity of changes across different age intervals, these genes were categorized into four distinct patterns. Class I represented stable expression types, showing gradual long-term upregulation or downregulation without pronounced fluctuations, as exemplified by PODXL2 (Fig 5A). Class II comprised early-life changing types, which exhibited upregulation or downregulation during young adulthood before gradually stabilizing, such as SNRNP70 (Fig 5B). Class III included late-life accelerating types, characterized by relative stability during young and middle-aged stages followed by sharp increases or decreases around age 60, as seen in USP17L2 (Fig 5C). Class IV consisted of mid-life fluctuating types, typically displaying inflection points with initial increases followed by decreases or vice versa around the transition to middle age or elderly stages, exemplified by EDA2R (Fig 5D).

Importantly, using first-derivative analysis of the GAM-fitted trajectories, we identified a dominant transition window at approximately 35 years and a secondary window at approximately 70 years (Fig 5E), suggesting that these age points may represent critical windows for transcriptional trajectory change in MASLD. Furthermore, GAM results revealed differences in trajectory morphology between MASLD and controls. For instance, USP17L2 exhibited late-life accelerating upregulation in MASLD, whereas in controls it followed a relatively stable declining trend (Fig 5C). Overall, MASLD demonstrates distinct and diverse age-related expression trajectories.

### Functional enrichment analysis of age-associated MASLD genes

**Fig 6:**
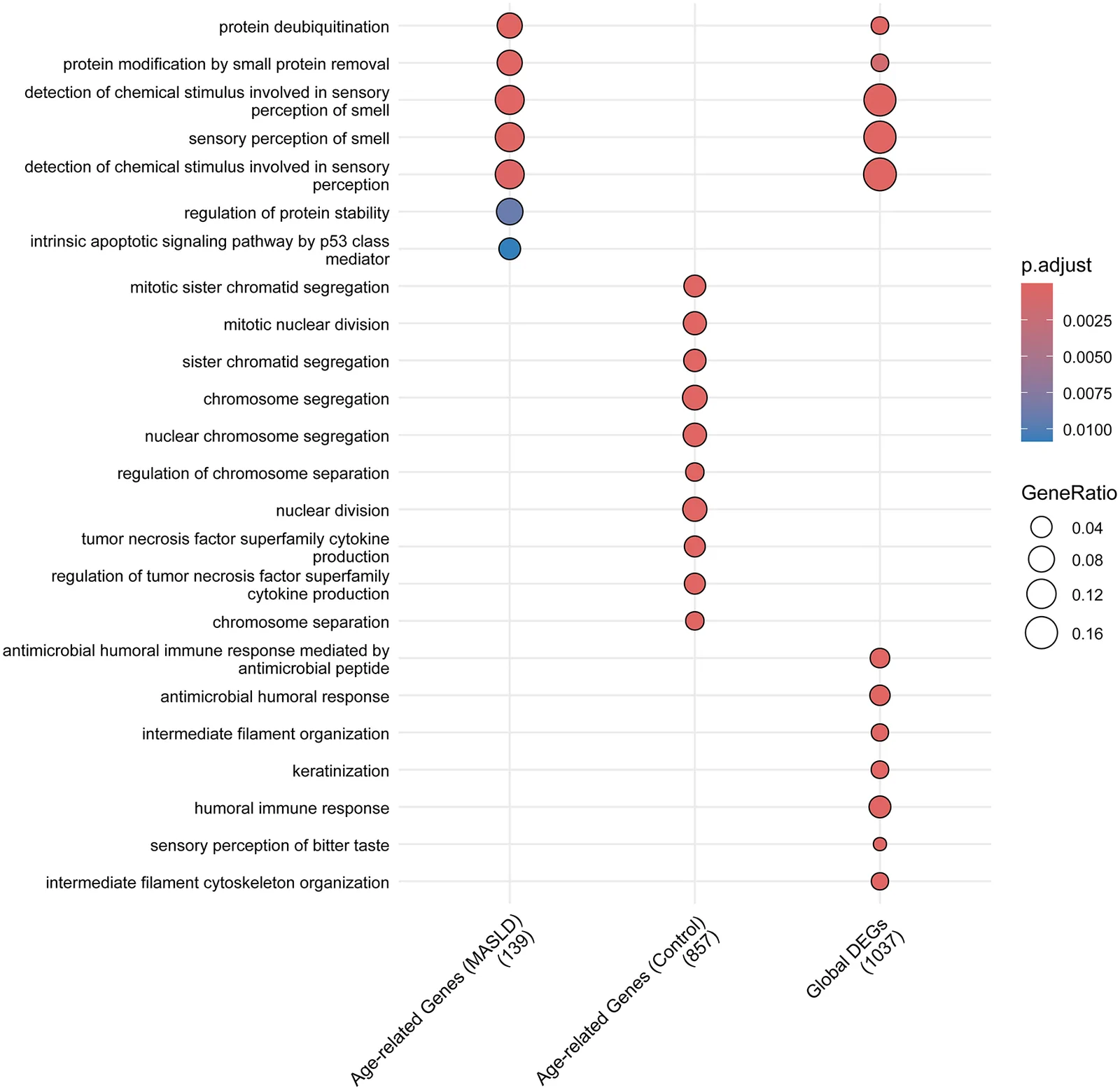
Comparative GO enrichment across gene sets. The horizontal axis of the GO bubble plot represents the three gene sets. The vertical axis displays the top 10 enriched GO biological process terms.

To further investigate the biological functions of age-associated genes in MASLD, we performed GO-BP enrichment analysis on three sets of genes including age-associated genes in the MASLD group, age-associated genes in the control group, and global DEGs between MASLD and controls. To observe the most significant results, we examined the top 10 enriched pathways of each set based on *padj* values. Results showed that physiological aging-related genes in the control group were primarily enriched in classical cell cycle regulation and chromosome segregation or stability-related processes (such as mitotic nuclear division and sister chromatid segregation). This suggests that during natural liver aging, mechanisms associated with proliferation and cell division are preferentially enriched (Fig 6A).

Notably, age-associated genes in MASLD exhibited a functional profile distinct from physiological aging. These genes were significantly enriched in protein deubiquitination and processes involved in protein modification removal, accompanied by enrichment of p53-associated stress and apoptotic signaling terms. These findings indicate that age-associated transcriptional changes in MASLD are functionally distinct from those observed in physiological aging. Rather than being primarily enriched in cell cycle-related processes as seen in controls, MASLD age-associated genes more preferentially involve pathways related to protein quality control and p53-mediated stress responses. Additionally, certain sensory perception-related terms, such as sensory perception of smell, appeared simultaneously in both age-associated MASLD genes and global DEGs (Fig 6A).

Global DEGs showed a lot overlap with the other two gene sets. Furthermore, they were enriched in processes such as humoral immune response and cellular structural changes, including intermediate filament organization, which aligns with the characteristic immune inflammation background and tissue structural alterations in MASLD (Fig 6A).

In summary, comparative enrichment analysis across the three gene sets suggests that age-related transcriptional changes in MASLD exhibit disease-specific and age-dependent molecular remodeling characteristics, preferentially involving pathways related to protein homeostasis and stress regulation.

### Associations of age-associated MASLD genes with histological severity

**Fig 7:**
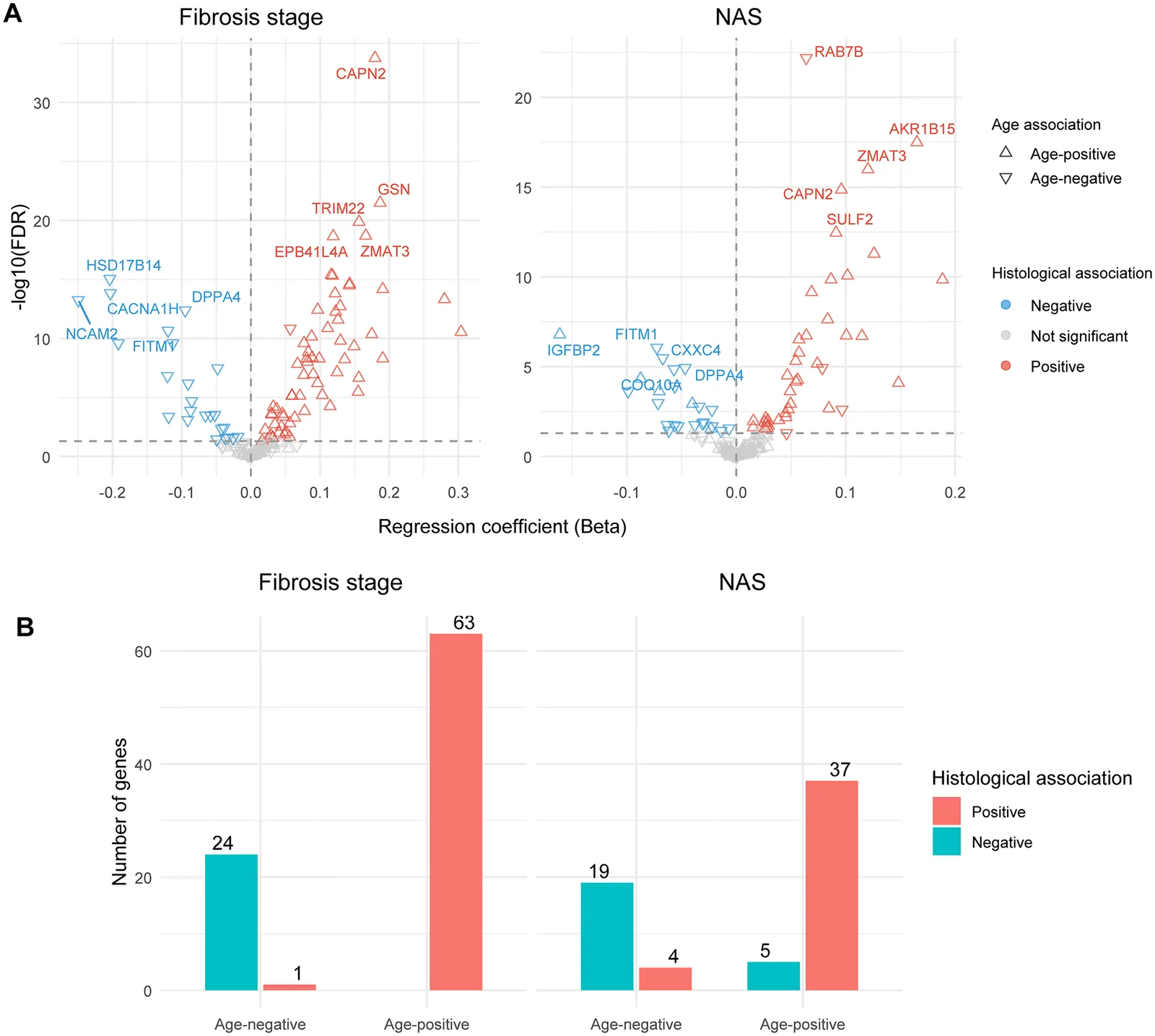
Associations of age-associated MASLD genes with histological severity. (A) Associations of MASLD age-associated genes with fibrosis stage and NAS in MASLD. (B) Numbers of genes grouped by age-related direction and histological association.

To evaluate the pathological associations of the 180 MASLD age-associated genes, we assessed their associations with fibrosis stage and NAS after adjustment for age and dataset source (Fig 7A). Specifically, 88 genes were significantly associated with fibrosis stage and 65 with NAS (FDR < 0.05). Most fibrosis associations were concordant with age-related trends. 63 genes were positively associated with both age and fibrosis, whereas 24 genes were negatively associated with both. Only one gene was negatively associated with age but positively associated with fibrosis. For NAS, 37 genes were positively associated with both age and NAS, and 19 genes were negatively associated with both, whereas 9 genes showed opposite age- and NAS-related trends. In addition, some age-associated genes were significantly associated with both fibrosis stage and NAS. For example, ZMAT3 showed positive associations with both measures, whereas FITM1 showed negative associations with both. These findings indicate that most MASLD age-associated transcriptional changes are aligned with increasing histological severity, particularly fibrosis progression.

## Discussion

MASLD is a chronic liver metabolic disorder. In this context, age is a well-established risk factor for MASLD progression [23], yet studies of transcriptomic changes associated with aging in MASLD across the full lifespan remain limited. In this study, by collecting multi-center liver transcriptomic data, we integrated a cross-age cohort and employed DEA, age correlation analysis, and GAM-based nonlinear modeling to construct expression trajectories of MASLD ages 9–92 years. Overall, although aging provides a biological context that increases susceptibility to MASLD [5,12,23], our results indicate that age-related transcriptional changes in MASLD showed limited overlap with normal (physiological) aging yet diverged markedly at the functional level.

Among the 180 age-correlated genes identified in MASLD, only 22 overlapped with age-correlated genes identified in the control group, and some overlapping genes displayed opposite trends between the two groups, such as BHLHA9. This result suggests that age-related alterations in MASLD do not simply represent an amplification of physiological aging but rather reflect a MASLD-specific deviation under chronic metabolic burden [12,25]. GO enrichment analysis further supports this notion. Age-correlated genes in the control group were primarily enriched in pathways such as cell cycle regulation and chromosome segregation, aligning with classical aging hallmarks associated with tissue renewal or proliferative changes [8,26,27]. In contrast, age-correlated genes in MASLD were more concentrated in protein deubiquitination, homeostasis regulation, and p53-mediated stress and apoptotic pathways. This functional divergence may partially explain the limited overlap of age-associated genes between the MASLD and control groups.

Mechanistically, we speculate that, with aging, chronic metabolic dysregulation places persistent stress on proteostasis. Under sustained demand on the ubiquitin-proteasome system, autophagy, and chaperone pathways, the upregulation of deubiquitination and p53-mediated stress and apoptosis related mechanisms may suggest that the liver is changing toward balancing homeostatic maintenance with damage removal during MASLD progression [41,49–51]. It may help explain why older patients with MASLD are at higher risk of advanced fibrosis and hepatocellular carcinoma, consistent with disease-specific disruption of proteostasis and stress responses layered onto normal aging, rather than MASLD representing accelerated aging per se.

Linear correlation analysis assumes a constant age effect and may miss stage-specific transitions [37,42,48]. Therefore, we used GAM model for nonlinear trajectory fitting and identified four predominant patterns, including stable expression, early-life change, late-life acceleration, and mid-life fluctuation. These four patterns may reflect distinct biological phases in MASLD, corresponding to core MASLD features that are relatively age-independent, early susceptibility establishment or metabolic dysregulation onset, midlife transitional metabolic adjustments, and late-life risk amplification, respectively. We need to emphasize that not all age-correlated genes in MASLD fell into these four patterns, but they accounted for the majority of observed trajectory changes.

Notably, inflection points were enriched around ~35 and ~75 years. These ages reflect statistically supported inflection windows from the GAM model rather than clinical staging or intervention thresholds, suggesting that MASLD transcriptomes may undergo phase-dependent alternations across the lifespan [52,53]. Around age 35, certain genes exhibited early changes followed by stabilization, such as SNRNP70, potentially corresponding to a transition from higher metabolic plasticity to gradual accumulation of chronic metabolic stress [11,52]. Around age 60, late-accelerating genes, such as USP17L2, showed more pronounced changes in MASLD without similar trends in controls, suggesting greater accumulation of MASLD-associated homeostatic imbalance at older ages [6]. This pattern may reflect increased vulnerability in maintaining hepatocyte homeostasis among elderly individuals with MASLD and is consistent with the natural history of MASLD [54].

Ubiquitin-specific protease family members, including USP17L2, USP17L3, and USP17L8, were positively correlated with age in MASLD and were generally upregulated relative to controls. These highly homologous USP17-family deubiquitinases [55] contributed to the enrichment of protein deubiquitination and protein stability-related pathways. USP17 stabilizes SET8 and suppresses p21-dependent senescence [56]. Aging promotes the accumulation of p21-positive senescent hepatocytes with impaired mitochondrial fatty-acid oxidation, which increases hepatic lipid deposition [41]. The age-related induction of USP17L genes may therefore represent a compensatory response that limits hepatocyte senescence. USP17 also stabilizes c-Myc and enhances glycolysis [57]. In hepatic stellate cells, c-Myc-dependent glycolytic reprogramming promotes cellular activation and liver fibrosis [58]. USP17 also deubiquitinates p62 and regulates lysosomal trafficking [59]. This process may be relevant to MASLD because saturated fatty acids impair autophagosome–lysosome fusion and cause the accumulation of p62 and ubiquitinated proteins in hepatocytes [60]. USP15 provides further evidence that deubiquitinases can promote hepatic lipid accumulation by stabilizing FABP1, FABP4, PLIN1, and PLIN2 [61]. These findings suggest that USP17L2, USP17L3, and USP17L8 may connect aging with MASLD through SET8–p21-dependent senescence, c-Myc-driven glycolytic activation of hepatic stellate cells, and p62-related lysosomal dysfunction. Prior studies have shown that USP9X-mediated deubiquitination of NRP1 enhances hepatic stellate cell activation and promotes liver fibrosis [62], and that loss of the deubiquitinase USP15 ameliorates MASLD [63]. Together, these observations support the view that age-associated upregulation of USP family members may increase deubiquitination activity and proteostasis burden in MASLD, potentially contributing to fibrosis progression [64].

In addition, sensory perception-related pathways, particularly olfactory perception, were enriched in both global DEGs and age-associated genes. This finding may point to changes in hepatic chemosensory-related mechanisms in MASLD and is consistent with evidence that ectopic olfactory receptors can function outside the olfactory epithelium [65,66].

Importantly, clinicopathological analysis showed that age-related transcriptional changes were largely concordant with disease severity. Most age-positive genes were positively associated with fibrosis stage or NAS, whereas most age-negative genes showed negative associations. This concordance was strongest for fibrosis, indicating that age-related transcriptional remodeling in MASLD is closely linked to pathological progression. Genes such as ZMAT3 and FITM1, which were associated with both fibrosis stage and NAS, may represent candidate markers of age-related MASLD severity. Limitations remain in our study. First, because our analysis is based on retrospective, cross-sectional data, the turning age points identified by GAM describe overall patterns in the cohort rather than the course of disease in an individual over time and therefore need confirmation in longitudinal studies. Second, clinical metadata were not completely consistent across all cohorts. Although batch correction was applied, residual differences between studies may still have influenced the results. Finally, our interpretations are derived from transcriptomic data. Additional validation at the protein level, together with functional and cell type-specific analyses, will be necessary to further clarify the underlying mechanisms.

## Conclusion

Our findings indicate that although age is an important risk factor for MASLD, age-related transcriptomic changes in MASLD are clearly different from physiological liver aging. These age-correlated genes in MASLD are closely related to protein homeostasis, deubiquitination, and sensory perception pathways. Using nonlinear models, we not only identified four major patterns of transcriptional changes across the lifespan of MASLD patients, but also identified turning points around ages 35 and 75. This helps direct where to look for age-related mechanisms in MASLD and offers age-related clues for precision management in the clinic.

## Supporting information

S1 Table Metadata of included samples. (CSV)

S2 Table Differentially expressed genes identified by DESeq2. (XLSX)
